# Use of Catheters in Hypertrophy of Prostate

**Published:** 1874-01

**Authors:** T. B. Curtis


### Our Exchanges.

*BOSTON MEDICAL AND SURGICAL JOURNAL—November*, 1873:

Use of Catheters in Hypertrophy of the Prostate—*By T. B. Curtis, M.D.*—The object of this paper is to draw attention to certain practical points relating to the treatment to be used in cases of enlarged prostate, and particularly to set forth the value, in such cases, of the *vulcanized India-rubber catheter.* This instrument, which has long been in general use in Paris, is comparatively little used or known in Great Britain and in this country, and its many advantages are certainly underrated.

The vulcanized India-rubber instrument seems, if we may judge by the practice now prevalent in the Paris hospitals, and particularly in the Civiale ward of the Necker Hospital, to be of all catheters the most suitable for cases where retention is due to loss of power of the bladder, or to increase of volume of the prostate, and for cases where difficulty of catheterism results from change of direction of the urethra rather than from narrowing of its caliber, or the existence of false passage.

These catheters are of all sizes, from No. 12 of the French scale upwards, but by far the most useful are those comprised between Nos. 16 and 20; they are perfectly cylindrical, with a rounded end and an even, smooth surface. The instrument possesses the following qualities: First. It is perfectly supple—so much so that it can be easily wound round the finger. Second. It is not acted upon by the urine or by the secretions of the mucous membrane; it neither acquires a phosphatic crust, as the gum catheters rapidly do when tied in, nor does its surface in any way become deteriorated, even by a prolonged sojourn in the urethra; whereas, the black gum catheter is found, in a very few days, or even hours, to become so rough by disintegration of its coating as to necessitate removal. The perfect flexibility of the instrument is the cause of the facility with which it finds its way, of itself, so to speak, into the bladder, requiring no guidance, but only a succession of short, quick pushes, which propel it along the urethra; if any obstacle is encountered by the bluntly rounded tip, the pliable shaft, immediately bending, ceases to transmit the forward impulsion. It is, therefore, absolutely impossible to inflict an injury with this instrument, or to create a false passage, however awkwardly it may be handled. Nor does its use require any skill or practice; it is only necessary that the instrument, well oiled, should be held with the finger tips quite near the meatus, so as to send in only a half-inch or so of the shaft at each push; at the same time the surgeon should keep the urethra well stretched, with the left hand, by firm traction upon the penis. It is a mistake to use this catheter with a stylet, as some authors recommend; this way of proceeding deprives the instrument of the valuable quality of pliability, which enables it to insinuate itself through a tortuous urethra, as it will often do in cases where catheterism with a silver instrument is so difficult as to baffle the patient efforts of a most skillful surgeon. Another advantage which accrues from the suppleness of this instrument is that it is of all catheters the one whose continued presence is most easily tolerated by the urethra; it causes much less discomfort to the patient than the gum catheter, and it is not so apt by its presence to set up prostatitis or cystitis, much less to occasion sloughing of the floor of the urethra at the sub-pubic bend; it is well known that the more rigid gum catheter sometimes, in this way, causes perforation of the wall of the urethra, thereby producing a very troublesome fistula, which necessitates a plastic operation of difficult performance and uncertain issue. The second quality of retaining an unimpaired surface, however long the instrument may be left in the urethra, is also a reason why it should be preferred for tying in.

